# Analysis of different performance times of the voiced trill technique in older women

**DOI:** 10.1590/2317-1782/20232021323en

**Published:** 2023-10-06

**Authors:** Bárbara de Faria Morais Nogueira, Ana Cristina Côrtes Gama, Flávio Barbosa Nunes, Maria Luiza Diniz, Adriane Mesquita de Medeiros

**Affiliations:** 1 Departamento de Fonoaudiologia, Faculdade de Medicina, Universidade Federal de Minas Gerais - UFMG - Belo Horizonte (MG), Brasil.; 2 Programa de Pós-graduação em Ciências Fonoaudiológicas, Universidade Federal de Minas Gerais - UFMG - Belo Horizonte (MG), Brasil.; 3 Departamento de Oftalmologia e Otorrinolaringologia, Faculdade de Medicina, Universidade Federal de Minas Gerais - UFMG - Belo Horizonte (MG), Brasil.; 4 Departamento de Fonoaudiologia, Faculdade de Medicina, Universidade Federal de Minas Gerais - UFMG - Belo Horizonte (MG), Brasil.; 5 Programa de Pós-graduação em Ciências Fonoaudiológicas, Universidade Federal de Minas Gerais - UFMG - Belo Horizonte (MG), Brasil.

**Keywords:** Elderly, Voice, Aging, Voice Disorders, Voice Training

## Abstract

**Purpose:**

To analyze and compare the immediate vocal effects of the voiced trill technique in the assessment of acoustic and auditory-perceptual measures of older women with and without self-perceived vocal changes.

**Methods:**

Clinical, quasi-experimental study in older women, aged 60 to 70 years (n=53). A questionnaire on vocal self-perception, voice, and laryngeal assessment was applied, before and after performing the voiced trill technique. Before and during intervals of the technique, sustained vowel samples were collected, totaling four samples. Older women were divided into two groups: one with self-perceived voice changes (n=25), and the other without self-perceived voice changes (n=28). Auditory-perceptual assessments and acoustic analysis were performed. Statistical tests were used to correlate the data: ANOVA Test for repeated measures, Friedman Test, Wilcoxon Test, and Pearson's Chi-Square Test. For all tests, the significance level was set at 5%.

**Results:**

There was a predominance of moderate dysphonia in both groups, according to the auditory-perceptual judgment. There was no statistically significant difference between the groups in the assessment of the auditory-perceptual analysis regarding voice changes (improved, worsened, and unaltered voices) before and after the different technique performance times. Most older women improved their voice after 1 minute of performing the technique.

**Conclusion:**

Older women often have voice changes when considering the perceptual judgment of the voice. There was no scientific evidence as to the ideal time to obtain a better effect on older women's voices.

## INTRODUCTION

Maintaining good vocal quality enables older people to communicate effectively, have higher self-esteem, and remain engaged in their social groups^(1)^. With the increase in life expectancy among the population, greater attention should be given to their communication.

In the aging process, modifications can occur in the architecture of the phonatory system. This process is called presbylarynx, which results in physiological changes in voice production, known as presbyphonia^(2)^. However, presbyphonia may or may not be associated with presbylarynx. It is important to note that presbylarynx is common but not universal among older people, and it should not be confused with common characteristics of the larynx^(3)^. These modifications occur in the laryngeal cartilages, including loss of elasticity, decreased mobility of the laryngeal joints, accompanied by atrophy of the vestibular folds, changes in the vocal fold cover^(4)^, and so forth.

The natural aging of the voice, called presbyphonia, begins and develops depending on the individual's physical and psychological health and life history^(5)^. Vocal speech therapy in aging seeks to compensate for the characteristics of presbyphonia and to slow down the deterioration process triggered by age, improving the quality of life of the subject in everyday activities^(6)^.

The voice trill technique stands out among those constantly used in speech therapy. This tongue or lip vibration technique is classified as a semi-occluded vocal tract exercise, as it softens the contact between vocal folds, increases vocal resistance, optimizes mucosal wave movement, balances sub and supra-glottic pressures, reduces the risk of phonatory trauma, expands the vocal tract, and stimulates resonance^(5)^.

However, little is known about the effect of vocal exercise and the time required to perform it among older people, given their anatomical and physiological changes.

The interest in addressing presbylarynx and presbyphonia is due to the growing older population in recent years, as well as the need to expand studies related to this age group.

The present study aimed to analyze and compare the immediate vocal effects of the voiced trill technique in assessing acoustic and auditory-perceptual measures of older women with and without self-perceived vocal changes.

## METHODS

This clinical quasi-experimental study was approved by the Research Ethics Committee, according to evaluation report number 83004518.5.0000.5149. All participants were informed about the study and signed an informed consent form to participate in the research. The sample consisted of 53 socially active older women, aged 60 to 70 years.

Participants were selected based on the following inclusion criteria: being female; being 60 years or older; not having undergone prior speech therapy treatment for the voice in the past 12 months, thus excluding the possibility of participants having any vocal conditioning due to training with speech therapy techniques. The exclusion criteria were the inability to perform trill sounds and a diagnosis of laryngeal changes unrelated to presbylarynx.

The research consisted of administering a questionnaire on sociodemographic and vocal self-perception data, auditory-perceptual judgment, acoustic voice quality assessment, and laryngeal evaluation before and immediately after 1, 3, and 5 minutes of performing the voiced trill technique. The study was conducted at a public university’s Observatory of Functional Health in Speech Therapy.

The questionnaire included items on sociodemographic data such as age, educational attainment, and the Screening for Voice Disorders in Older Adults- RAVI^(7)^.

RAVI is a short, low-cost, easily administered, self-reported questionnaire that allows screening for vocal changes in older people. It has 10 questions, each with three response options (no, sometimes, and always), on a Likert-type scale graded from zero to two, as follows: no = 0, sometimes = 1, and always = 2. The sum of all answered questions was calculated to analyze the responses. When the final score was above 2 points, it indicated the presence of vocal changes, while a score equal to or lower than 2 points indicated the absence of vocal changes^(7)^. Hence, RAVI was used as a screening tool to help determine the prevalence of voice disorders in older adults^(7)^.

Before and during technique performance intervals, samples of the sustained vowel /a/ were collected, totaling four samples. The first sample corresponds to the technique pre-performance moment (M0), and the second sample was taken after 1 minute (M1). Then, the technique was performed for another 2 minutes, reaching a total of 3 minutes (M2), and yet another 2 minutes, completing 5 minutes (M3) of performing the voiced trill technique.

All participants produced the sustained vowel /a/ at their maximum phonation time with the usual voice frequency and intensity. They only started the technique sequence after the evaluator demonstrated how to do it, followed by a brief training session with one or two correct repetitions of the voiced trill technique performed by participants. Before collecting M0, the older adults rested their voices for at least 30 seconds.

For speech material collection, the participants remained seated in a quiet environment throughout the entire period, and they were instructed to perform the voiced trill at their maximum phonation time, using their usual pitch without variation, for 1, 3, and 5 minutes. If a participant was unable to perform tongue trill, it was substituted with lip trill (n = 17). Throughout the entire duration of the vocal technique, participants were allowed to consume up to 250 ml of water to moisten the oral cavity, which may become dry due to the vibrations performed during the exercise. It is worth noting that hydration does not interfere with the results, as systemic hydration takes a few hours to reach the larynx^(8)^.

Exercise performance was recorded in the CSL program from KayPentax®️, model 4,500, connected to a professional unidirectional microphone, Shure®️ brand, model SM48-LC, positioned 10 cm from the speaker, in a silent environment with room noise level below 50 dB SPL (verified with a sound pressure level meter manufactured by Radio Shack®️). Sustained vowel /a/ samples were used for auditory-perceptual and acoustic evaluations.

The otorhinolaryngological evaluation was conducted by an otorhinolaryngologist at a university hospital within 15 days of questionnaire administration and technique performance. Videolaryngoscopy was performed using a 70° telescope manufactured by Storz®️, a 300-watt xenon light source (Storz®️), and a telecam DX microcamera (Storz®️). All participants were asked to emit the sustained vowel /i/ for at least 2 seconds at their usual frequency.

The examining physician completed the Reflux Finding Score^(9)^, validated for Brazilian Portuguese as the Laryngeal Findings Scale, which scores laryngeal inflammatory findings and signs compatible with laryngopharyngeal reflux (LPR) based on videolaryngoscopy examination. The presence of subglottic edema, ventricular obliteration, erythema or hyperemia, vocal fold edema, diffuse laryngeal edema, interarytenoid region hypertrophy, granuloma/granulation tissue, and thick endolaryngeal mucus was evaluated. A final score equal to or greater than seven points in the final sum indicates a high probability of LPR^(9)^. None of the participants had LPR considering the instrument's cutoff score. All examination information was recorded in a medical report and provided to the participants at the end. The researcher also received a copy of each participant's examination.

**Figure 1:**
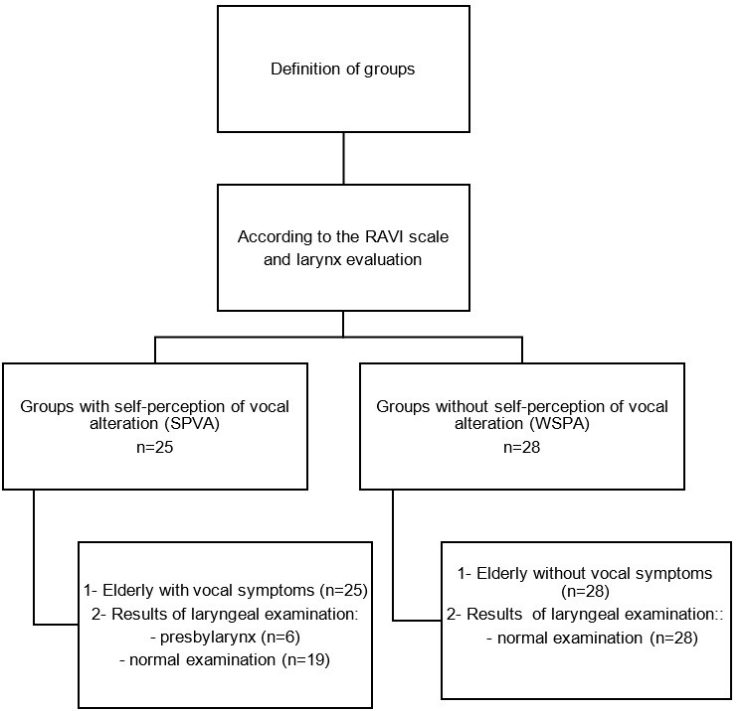
Flowchart of the definition of comparison groups

The study groups were defined after collecting all data. Participants were divided into two groups based on the RAVI classification^(7,10,11)^ and the laryngeal examination results: a group of older women with self-perceived vocal changes, vocal symptoms, and laryngeal examination findings consistent with older laryngeal characteristics (SPVA/n=25), and a group of older women without self-perceived vocal changes, without vocal symptoms, and with a normal laryngeal examination (WSPA/n=28) (Figure 1).

Auditory-perceptual evaluations and acoustic analysis were performed to analyze the outcomes of the vocal technique. Three speech-language-hearing pathologists with at least 5 years of experience in the field conducted these evaluations. The judges received randomized voice recordings on a CD and were instructed to listen to the recordings as many times as necessary in a silent environment. The analysis was based on the sustained vowel /a/. For each pair of voices, the judges were instructed to determine if the second voice improved, worsened, or remained unchanged. When the judge observed a change in the voice (improvement or deterioration), they were asked to identify the auditory-perceptual parameter (overall grade of dysphonia - G) from the GRBASI scale that most correlated with the change in voice. It should be noted that the voices were evaluated considering time “0” as the absence of vocal deviation. The judges had no prior knowledge of whether the analyzed voice was pre- or post-exercise, the duration of the exercise, or the participants' ages.

For acoustic evaluation of the voices, the following acoustic parameters were analyzed using the CSL program (KayPentax®️): average fundamental frequency (f0) in Hz, jitter (%), shimmer (%), pitch perturbation quotient (PPQ) (%), amplitude perturbation quotient (APQ) (%), and harmonic-to-noise ratio (HNR) in dB. The initial /a/ emission vocal onset was eliminated for analyses, and from that point, a time of 3.5 seconds was considered as the standard analysis window, discarding the rest(8). The program's manual indicates the following normal values of acoustic measures: f0 243.97 Hz, jitter 0.63%, shimmer 1.99%, PPQ 0.36%, APQ 1.39%, and HNR 0.11 dB^(12)^.

Cepstral measures were also extracted, including CPP (Cepstral Peak Prominence) and CPPs (Cepstral Peak Prominence Smoothed), which correspond to the logarithmic scale Fourier transform^(13)^.

The software used was Praat, version 6.1.16, employing parameters described by Phadke et al.^(14)^. The task was recorded using the aforementioned equipment, and the beginning and end of the recording were excluded to avoid interfering with the cepstral analysis of the acoustic signal.

The analysis of variables initially involved descriptive statistics, including relative and absolute frequency distribution of categorical variables and summary statistics of numerical variables. Intra-rater agreement for auditory-perceptual evaluation was assessed using the AC1 statistic in R software version 3.2. The following agreement values were found: evaluator one: 0.517; evaluator two: 0.434; evaluator three: 0.458. These values indicate moderate agreement among the three evaluators^(15)^.

Based on the distribution analysis of quantitative variables using the Kolmogorov-Smirnov test, statistical tests were defined to compare the acoustic parameters between groups and across different technique performance times (repeated measures ANOVA, Friedman test, and Wilcoxon test). The statistical tests used to assess the association between auditory-perceptual judgments and the SPVA and WSPA groups were Fisher's exact test and Pearson's chi-square test. The significance level was set at 5%.

## RESULTS

Most participants in this study were 60 to 70 years old (60.37%), literate (75.47%), and retired (88.67%). Table 1 shows a predominance of moderate dysphonia in both comparison groups, according to the specialists’ auditory-perceptual judgments.

**Table 1:** Description of the general degree of dysphonia in the perceptual-auditory assessment of the groups prior to the implementation of voiced vibration technique

**Groups**	**General Degree**	**n (%)**
SPVA (n=25)	Neutral	3 (12.0)
	Light	5 (20.0)
	Moderate	13 (52.0)
	Severe	4 (16.0)
WSPA (n=28)	Neutral	3 (10.7)
	Light	10 (35.7)
	Moderate	12 (42.9)
	Severe	3 (10.7)

In the auditory-perceptual assessment, there was a predominance of voice improvement after performing the technique in all time intervals, with a higher frequency observed after 1 minute of performing it. When comparing the groups (Table 2) regarding the participants' self-perceived vocal changes, there was no statistically significant difference in the association of voice improvement, deterioration, or unchanged voices. In other words, the change in voice after performing the technique in all time intervals was not different between older individuals with and without self-perceived vocal changes.

**Table 2:** Association of auditory-perceptual assessment between SPVA and WSPA according to the time of execution of the technique

**Auditory-perception Assessment**		**Total sample n (%)**	**Groups**		**P value** 1
			SPVA **(n=25)**	WSPA **(n=28)**	
			**n (%)**	**n (%)**	
G after 1 minute	Improved	30 (56.6%)	13 (52.0)	17 (60.7)	0.747*
	Worsed	9 (17.0%)	4 (16.0)	5 (17.9)	
	Unaltered	14 (26.4%)	8 (32.0)	6 (21.4)	
G after 3 minutes	Improved	25 (47.1%)	12 (48.0)	13 (46.4)	0.828**
	Worsed	13 (24.5%)	7 (28.0)	6 (21.4)	
	Unaltered	15 (28.3%)	6 (24.0)	9 (32.1)	
G after 5 minutes	Improved	23 (43.4%)	13 (52.0)	10 (35.7)	0.416**
	Worsed	13 (24.5%)	6 (24.0)	7 (25.0)	
	Unaltered	17 (32.0%)	6 (24.0)	11 (39.3)	

1 Significant p-value ≤ 0.05;

* Fisher's exact test;

** Pearson's Chi-Square test

Caption: G = general grade of GRBASI; SPVA = group with self-perceived vocal alteration; WSPA = group without self-perceived vocal alteration

Table 3 compares the acoustic parameters with the performance times of the voiced trill technique. In the SPVA group, a statistically significant association was observed only for APQ (p = 0.022). The Wilcoxon test was conducted to determine which pair(s) showed the difference, revealing it between M0 and M1 (p = 0.013). The remaining associations were not statistically significant.

**Table 3:** Comparison of the acoustic parameters of the group with self-perceived vocal alteration (SPVA) between the execution times of the technique (n=25)

**Variables**	**Moment**	**Mean**	**Median**	**SD**	**P value**
F0 (Hz)^1^	M0	188	186.06	31.67	0.878
	M1	191.76	189.72	43.88	
	M2	190.12	191.89	43.19	
	M3	191.76	194.85	44.33	
APQ^2^	M0^3^	5.37	4.53	3.06	0.022*
	M1^3^	4.02	3.84	2.34	
	M2	4.8	4.02	3.04	
	M3	4.97	3.8	4.43	
PPQ^2^	M0	1.38	1.01	1.02	0.123
	M1	0.94	0.65	0.82	
	M2	1.01	0.82	0.74	
	M3	1.17	0.86	1.09	
HNR^2^	M0	0.23	0.19	0.1	0.231
	M1	0.18	0.17	0.06	
	M2	0.18	0.17	0.05	
	M3	0.21	0.16	0.1	
SHIMMER^2^	M0	7.7	6.79	4.53	
	M1	5.74	4.84	3.84	0.113
	M2	6.57	5.02	5.47	
	M3	6.74	5.46	5.24	
JITTER^2^	M0	2.37	1.88	1.69	0.311
	M1	1.87	1.14	1.67	
	M2	1.72	1.43	1.2	
	M3	2.02	1.33	1.88	
CPP^1^	M0	26.87	27.35	3.37	0.369
	M1	27.53	27.79	2.76	
	M2	27.48	27.49	3.05	
	M3	27.4	27.76	3.2	
CPPs^1^	M0	15.98	15.96	2.84	0.124
	M1	16.87	16.96	2.32	
	M2	16.53	16	2.9	
	M3	16.62	16.67	2.61	

1 Anova test for repeated measurements;

2 Friedman's test;

3 Wilcoxon Test;

* Significant p-value ≤ 0.05

Caption: SD = standard deviation; F0 = fundamental frequency; Hz = hertz; APQ = amplitude perturbation quotient; PPQ = pitch perturbation quotient; HNR = harmonics-to-noise ratio; CPP = cepstral peak prominence; CPPs = smoothed cepstral peak prominence; M0 = evaluation before performing the technique; M1 = after 1 minute of technique execution; M2 = after 3 minutes of technique execution; M3 = after 5 minutes of technique execution

The same analyses as in Table 3 were performed in Table 4, but now for the WSPA group. It shows no statistically significant association in any of the analyses.

**Table 4:** Comparison of the acoustic parameters of the group without self-perceived vocal alteration (WSPA) between the execution times of the technique (n=28)

**Variables**	**Moment**	**Mean**	**Median**	**SD**	**P value** *
F0 (Hz)^1^	M0	177.62	182.55	32.73	0.262
	M1	186.45	189.53	29.63	
	M2	179.83	183.01	36.44	
	M3	184.64	185.49	28.34	
APQ^2^	M0	4.39	3.64	2.41	0.478
	M1	3.16	1.76	1.4	
	M2	4.13	2.94	2.85	
	M3	3.54	2.95	1.72	
PPQ^2^	M0	0.99	0.67	0.89	0.887
	M1	0.69	0.53	0.5	
	M2	0.83	0.61	0.74	
	M3	0.82	0.52	0.73	
HNR^2^	M0	0.19	0.16	0.08	0.219
	M1	0.16	0.14	0.05	
	M2	0.17	0.15	0.07	
	M3	0.17	0.15	0.06	
SHIMMER^2^	M0	6.06	5.3	3.6	0.372
	M1	4.25	3.79	1.86	
	M2	5.89	3.97	4.29	
	M3	4.94	4.05	2.65	
JITTER^2^	M0	1.76	1.2	1.5	0.663
	M1	1.22	0.96	0.81	
	M2	1.47	1.13	1.26	
	M3	1.41	0.87	1.13	
CPP^1^	M0	27.51	27.5	3.02	0.212
	M1	28.51	28.48	1.98	
	M2	28.36	28.54	2.93	
	M3	28.65	29.2	2.63	
CPPs^2^	M0	16.49	16.57	2.67	0.096
	M1	17.65	17.5	1.71	
	M2	17.29	17.01	2.69	
	M3	17.64	18	2.3	

1 Anova test for repeated measurements;

2 Friedman's test;

* Significant p-value ≤ 0.05

Caption: SD = standard deviation; F0 = fundamental frequency; Hz = hertz; APQ = amplitude perturbation quotient; PPQ = pitch perturbation quotient; HNR = harmonics-to-noise ratio; CPP = cepstral peak prominence; CPPs = smoothed cepstral peak prominence; M0 = evaluation before technique execution; M1 = after 1 minute of technique execution; M2 = after 3 minutes of technique execution; M3 = after 5 minutes of technique execution

## DISCUSSION

In the present study, there was a predominance of moderate dysphonia, regardless of the group examined, based on the specialists’ auditory-perceptual evaluation. This finding corroborates a study^(16)^ that assessed the vocal characteristics of institutionalized older individuals to determine whether these characteristics would interfere with communication and speech patterns. The authors concluded that the predominant degree of vocal change was moderate and that there are age-related changes in voice parameters. It is known that natural voice aging is expected with advancing age^(5)^, and the study's results reinforce the absence of a correlation between the presence of presbyphonia and presbylarynx.

The specialist's auditory-perceptual evaluation differs from the older people’s self-perception of vocal changes. A study evaluated the voices of a group of older individuals and related vocal quality to the degree of voice changes. This association was conducted through auditory-perceptual evaluation and a vocal self-assessment scale. The participants evaluated their own voices with a positive impact on their personal, professional, and social lives. The authors noted that even in voices with altered vocal quality, the results obtained were positive, with a predominance of good and excellent ratings in the vocal self-assessment by the older individuals^(17)^.

However, older adults with presbyphonia may have a hoarse, weak, breathy voice, along with reduced maximum phonation time and phonatory range. These characteristics, which are expected in senescence, can lead to speech unintelligibility and have a negative impact on the quality of life^(18)^. The vocal evaluation of the WSPA group, with older women without reported vocal symptoms, showed the presence of vocal changes in almost all participants. The lack of perception by older individuals regarding vocal symptoms may be related to some individuals having a positive self-image of their voice, while others may never have thought about it throughout their lives, and yet others may consider vocal symptoms as natural consequences of aging that cannot be improved. It is worth noting that the impact of vocal changes on the quality of life (which is an important aspect in the multidimensional assessment of voice) was not investigated in this study.

Research studies^(19-21)^ on tongue trill exercises were conducted in the adult population with and without vocal changes. It is understood that there are differences in vocal tract anatomy between adults and older adults^(8,16)^. Despite the absence of intergroup statistical significance in all vocal technique performance times, the present study found an improvement in older women’s vocal quality. It can be said that the effect of the technique was positive in all three evaluated times in both groups, but the degree of improvement decreased as the performance time increased. This result was not expected since for a better physiological adaptation to the laryngeal condition of older adults, they might require longer duration vocal exercise stimulation. It is worth noting that almost all participants had a normal larynx. Respiratory measures were not evaluated, but they may have interfered with the balance of vocal production dynamics, decreasing technique performance over time.

In a literature review^(22)^ on evidence of muscle hypertrophy in older individuals through resistance training, the authors showed that it is possible to improve muscle mass through resistance training in older individuals, especially in the oldest ones, as long as the exercises have the correct dosage, intensity, volume, and load. Little is known about this dosage in speech therapy treatment for older people’s voices. For the success of vocal therapy in older individuals, changes in vocal intensity, muscle use, and respiratory support are also necessary. Moreover, it is extremely important to maintain these changes in the phonatory system^(23)^.

The literature suggests that tongue trill exercises yield positive results starting from 3 minutes, and the ideal exercise performance time is 3 minutes for both sexes or three sets of 15 repetitions with 30-second intervals for adult women^(24)^. However, another study proposes a 5-minute performance for adult men^(20)^. In dysphonic children, however, it was found that tongue trill exercises did not result in improvements in any performance time (1, 3, 5, or 7 minutes)^(25)^.

A study^(5)^ was conducted in 33 individuals, with a mean age of 75 years, to observe the immediate effect of performing voiced oral airflow exercises with a semi-occluded vocal tract on their voices after 1 minute of exercise. It found that this exercise produces immediate positive effects on older people’s vocal quality, as observed in the perceptual analysis. This finding corroborates those in this research regarding the duration of the technique. It is worth noting that in both studies, the exercises involve a semi-occluded vocal tract, and that after 1 minute of exercise, older people’s vocal emission was better than their habitual vocal production.

This study analyzed the immediate effects of voiced trill exercises. Considering the longitudinal process of speech therapy follow-up, the literature describes the number of sessions, frequency, and duration of voice disorder treatment for older people, but the results are variable^(26)^. Some studies emphasize the benefit of intensive treatment for the older population^(27)^. It is estimated that this type of intensive approach promotes motor learning, behavior change, and better muscle performance in a shorter time compared to traditional therapy^(28)^.

Regarding the results of the acoustic analysis, there was a reduction in APQ, PPQ, PHR, shimmer, and jitter measures after the first minute of voiced trill exercises in both study groups, but with statistical significance only for APQ in SPVA. Although the found values decreased after the first minute, it can be said that they are altered since the program used is standardized for young women, aged 20 to 45 years^(29)^. The lack of reference values for the older population regarding the analyzed measures makes the interpretation of these data difficult.

There was a decrease in APQ values after performing voiced trill exercises, indicating greater stability in vocal fold vibration amplitude, cycle to cycle, and consequently better phonatory control. The decrease in APQ refers to the passage and control of transglottic airflow in each glottic cycle, suggesting less transglottic airflow escape^(30)^. This result indicates an improvement in older people’s sustained vowel emission, especially for those with self-perceived vocal changes. The reduction in some acoustic measures reinforces the initial positive effect of the technique on older women’s vocal quality in both groups after 1 minute, with some stability in the values after that time.

The analysis of cepstral measures as a function of exercise time did not demonstrate statistical significance, despite an increase in the values obtained for CPP and CPPs measures in both groups. Therefore, it is not possible to establish a temporal relationship between the duration of the employed technique and the improvement of vocal parameters highly correlated with cepstral measures, such as roughness and breathiness^(3)^. On the other hand, it should be considered that muscle fiber deterioration is one of the signs of aging^(4)^. Therefore, it is assumed that progressive exercise performance may have reached muscle fatigue, and consequently, there was no progressive functional improvement, as demonstrated in studies in younger individuals^(20,24)^.

It is important to consider age and sex when prescribing the duration of the voiced trill technique to achieve more immediate and positive voice results. Older people can benefit from vocal health promotion programs through guidance on vocal behavior and training in vocal techniques to reduce symptoms and improve vocal quality. Vocal therapy in aging aims to compensate for the characteristics of presbyphonia, optimize communicative aspects, and slow down the age-related deterioration process, improving the individual's quality of life in daily activities^(8)^.

The results of the auditory-perceptual evaluation and acoustic analysis in the present study demonstrate the benefit of the voiced trill technique for older women’s voices, particularly in the SPVA group after 1 minute of the performance. However, it is not possible to determine the ideal duration of the technique to improve vocal quality in older women.

We consider as a limitation of this study the lack of sample size calculation, the small sample size, and the convenience sampling method, which prevents the generalization of the results. The decision to compare the groups considering older women’s self-perception of vocal changes, along with the results of the laryngeal examination, highlights the complexity of conducting voice research in the older population, as they may be affected by various chronic diseases, medication use, and lifelong inappropriate vocal behaviors, which can be etiological factors for dysphonia. It was not evaluated whether performing different voiced trill techniques (using lips and tongue) interfered with the older women’s results in any way.

Dysphonia is known to have multiple causes, and when establishing its etiology in the older population, the peculiarities resulting from the aging process must be taken into account. Even though natural voice changes occur in older people, it is of utmost importance to clinically investigate their vocal symptoms with a comprehensive evaluation. Vocal symptoms in older people need to be valued and assessed, as they can be signs of diseases such as laryngopharyngeal reflux disease, laryngeal tumors, and early-stage neurodegenerative diseases. Presbyphonia diagnosis should consider the auditory-perceptual evaluation, acoustic analysis, self-perceived voice quality, and laryngeal assessment in the elderly.

It is worth noting that the older women studied in this research did not have laryngopharyngeal reflux. The acid composition can harm the larynx and cause inflammation, reducing the individual's communication effectiveness and can cause or exacerbate voice disorders^(1)^, which is important to investigate in the elderly population.

Thus, the results of this study can assist and guide clinical speech therapy practice in the older population, as the use of the voiced trill technique yielded positive results. This exercise can be used to improve the vocal quality of older women regardless of self-perceived vocal changes.

Further studies investigating the effects of vocal exercise duration in males are also necessary to understand the functional outcomes of vocal folds in different sexes and determine vocal technique performance times. Future research evaluating the immediate effects of the voiced trill technique with frequency variation is also important, considering the functional training aspects aimed at strengthening and improving muscle mobility in the older population.

Speech-language-hearing pathologists and voice clinicians should consider the recommendations regarding exercise prescriptions, especially in the older population, as they require knowledge about the physiology of aging. Therefore, it is necessary to analyze the immediate and long-term effects of exercises, considering the vocal technique performance time and number of daily repetitions.

## CONCLUSION

Older women often have vocal changes, considering experts’ auditory-perceptual judgment of the voice. Despite the results indicating a predominance of voice improvement in older women after performing the voiced trill technique in various durations, there was no scientific evidence regarding the ideal time to achieve a better effect on their voices. It is noteworthy that both investigated groups had a positive auditory-perceptual response after the first minute.

## References

[1] Gois AC, Pernambuco LA, Lima KC (2018). Factors associated with voice disorders among the elderly: a systematic review. Rev Bras Otorrinolaringol.

[2] Retuert DR, Olavarria CL, Frías ME, Ovalle RA (2017). Presbilaringe: revisión de la literatura. Rev Otorrinolaringol.

[3] Ziegler A, Abbott KV, Johns M, Klein A, Hapner ER (2014). Preliminary data on two voice theraphy interventions in the treatment of presbyphonia. Laryngoscope.

[4] Wilder CN (1978). Vocal aging..

[5] Siracusa MGP, Oliveira G, Madazio G, Behlau M (2011). Efeito imediato do exercício de sopro sonorizado na voz do idoso. J Soc Bras Fonoaudiol.

[6] Rocha TF, Amaral FP, Hanayama EM (2007). Extensão vocal de idosos coralistas e não coralistas. Rev CEFAC.

[7] Pernambuco LA, Espelt A, Lima KC (2017). Screening for voice disorders in older adults (RAVI) — Part III: cutoff score and clinical consistency. J Voice.

[8] Cielo CA, Lima JPM, Christmann MK (2016). Comparação dos efeitos do finger kazoo e da fonação em tubo em mulheres com voz normal. Audiol Commun Res.

[9] Almeida AG, Saliture TC, Silva AS, Eckley CA (2013). Translation and cultural adaptation of the Reflux Finding Score into brazilian portuguese. Rev Bras Otorrinolaringol.

[10] Pernambuco LA, Espelt A, Magalhães HV, Cavalcanti RVA, Lima KC (2016). Screening for voice disorders in older adults (Rastreamento de Alterações Vocais em Idosos — RAVI) — Part I: validity evidence based on test content and response processes. J Voice.

[11] Pernambuco LA, Espelt A, Costa EBM, Lima KC (2016). Screening for voice disorders in the elderly (Rastreamento de Alterações Vocais em Idosos; RAVI) — Part II: validity evidence and reliability. J Voice.

[12] Moreira FS, Gama ACC (2017). Efeito do tempo de execução do exercício vocal sopro e som agudo na voz de mulheres. CoDAS.

[13] Fraile R, Godino-Llorente JI (2014). Cepstral peak prominence: a comprehensive analysis. Biomed Signal Process Control.

[14] Phadke KV, Laukkanen AM, Ilomäki I, Kankare E, Geneid A, Švec JG (2020). Cepstral and perceptual investigations in female teachers with functionally healthy voice. J Voice.

[15] Landis JR, Koch GG (1977). The measurement of observer agreement for categorical data. Biometrics.

[16] Menezes LN, Vicente LCC (2007). Envelhecimento vocal em idosos institucionalizados. Rev CEFAC.

[17] Cassol M (2006). Avaliação da percepção do envelhecimento vocal em idosos. Estud Interdiscip Envelhec..

[18] Favoretto NC, Carleto NG, Arakawa AM, Alcalde MP, Bastos JRM, Caldana ML (2017). Portal dos idosos: desenvolvimento e avaliação de um website com informações sobre o processo de envelhecimento e as principais alterações fonoaudiológicas que acometem os idosos. CoDAS.

[19] Menezes MH, Ubrig-Zancanella MT, Cunha MG, Cordeiro GF, Nemr K, Tsuji DH (2011). The relationship between tongue trill performance duration and vocal changes in dysphonic women. J Voice.

[20] Menezes M, Duprat AC, Costa HO (2005). Vocal and laryngeal effects of voiced tongue vibration technique according to performance time. J Voice.

[21] Schwarz K, Cielo CA (2009). Modificações laríngeas e vocais produzidas pela técnica de vibração sonorizada de língua. Pro Fono.

[22] Pinheiro HA, Pereira LC, Santana FS, Alves AT, Fachin-Martins E, Karnikowski MGO (2018). Revisão treinamento de resistência para hipertrofia muscular em idosos. Fisioterapia Brasil.

[23] Fabron EMG, Silvério KCA, Berretin-Felix G, Andrade EC, Salles PF, Moreira PAM (2018). Voice therapy for the elderly with progression of intensity, frequency, and phonation time: case reports. CoDAS.

[24] Vasconcelos D, Gomes AOC, Araújo CMT (2016). Técnica de vibração sonorizada de lábios e língua: revisão de literatura. Distúrb Comun.

[25] Silva FC, Ramos LA, Souza BO, Medeiros AM, Gama ACC (2017). Tempo ideal de vibração sonorizada de língua em crianças disfônicas. Distúrb Comun.

[26] Godoy JF, Silverio KCA, Andrade EC, Brasolotto AG (2020). Método intensivo de terapia vocal para idosos. Audiol Commun Res.

[27] Lu F-L, Presley S, Lammers B (2013). Efficacy of intensive phonatoryrespiratory treatment (LSVT) for presbyphonia: two case reports. J Voice.

[28] Godoy J, Silverio K, Brasolotto A (2019). Effectiveness of vocal therapy for the elderly when applying conventional and intensive approaches: a randomized clinical trial. J Voice.

[29] Felippe ACN, Grillo MHMM, Grechi TH (2006). Normatização de medidas acústicas para vozes normais. Rev Bras Otorrinolaringol.

[30] Roman-Niehues G, Cielo CA (2010). Modificações vocais acústicas produzidas pelo som hiperagudo. Rev CEFAC.

