# Supplementary material for: Analysis of different performance times of the voiced trill technique in older women
Source: Codas. 2023 Oct 6;35(6):e20210323. doi: 10.1590/2317-1782/20232021323en (PMC10702712; doi:10.1590/2317-1782/20232021323en)
Supplement: Translation

# Translation: Análise dos tempos de execução da técnica de sons vibrantes em idosas

**DOI:** 10.1590/2317-1782/20232021323pt

**Authors:** Bárbara de Faria Morais Nogueira, Ana Cristina Côrtes Gama, Flávio Barbosa Nunes, Maria Luiza Diniz, Adriane Mesquita de Medeiros

**Affiliations:** 1 Departamento de Fonoaudiologia, Faculdade de Medicina, Universidade Federal de Minas Gerais - UFMG - Belo Horizonte (MG), Brasil.; 2 Programa de Pós-graduação em Ciências Fonoaudiológicas, Universidade Federal de Minas Gerais - UFMG - Belo Horizonte (MG), Brasil.; 3 Departamento de Oftalmologia e Otorrinolaringologia, Faculdade de Medicina, Universidade Federal de Minas Gerais - UFMG - Belo Horizonte (MG), Brasil.; 4 Departamento de Fonoaudiologia, Faculdade de Medicina, Universidade Federal de Minas Gerais - UFMG - Belo Horizonte (MG), Brasil.; 5 Programa de Pós-graduação em Ciências Fonoaudiológicas, Universidade Federal de Minas Gerais - UFMG - Belo Horizonte (MG), Brasil.

**Keywords:** Idoso, Voz, Envelhecimento, Distúrbios da Voz, Treinamento da Voz

## Abstract

**Objetivo:**

Analisar e comparar os efeitos vocais imediatos da técnica de sons vibrantes sonoros na avaliação de medidas acústicas e perceptivo-auditivas de idosas com e sem autopercepção de alteração vocal.

**Método:**

Estudo clínico quase experimental, envolvendo 53 idosas com idade entre 60 e 70 anos. Foi aplicado um questionário de autopercepção vocal, avaliação vocal e laríngea, antes e após a realização da técnica vocal com sons vibrantes sonoros. Antes e durante os intervalos da técnica, foram coletadas amostras de vogais sustentadas, totalizando quatro amostras. As mulheres idosas foram divididas em dois grupos: grupo com autopercepção de alteração vocal (n = 25) e grupo sem autopercepção de alteração vocal (n = 28). Foram realizadas avaliações perceptivo-auditivas e análise acústica. Os testes estatísticos foram utilizados para correlacionar os dados: Teste Anova para medidas repetidas, Teste de Friedman e Teste de Wilcoxon e Teste Qui-Quadrado de Pearson. Para todos os testes foi considerado nível de significância de 5%.

**Resultados:**

Houve predomínio de disfonia moderada em ambos os grupos, de acordo com a julgamento perceptivo-auditivo. Não houve diferença estatisticamente significante entre os grupos na avaliação da análise perceptivo-auditiva quanto à melhora, piora e voz inalterada antes e após os diferentes tempos de execução da técnica. A maioria das idosas melhorou a voz após um minuto de execução da técnica.

**Conclusão:**

As idosas apresentam elevada presença de alteração vocal quando considerada o julgamento perceptivo-auditivo da voz. Não houve evidências científicas quanto ao momento ideal para se obter um melhor efeito na voz das idosas.

## INTRODUÇÃO

A manutenção de uma boa qualidade de voz permite o idoso se comunicar eficazmente, ter maior autoestima e permanecer investido em seus grupos sociais^(^ 1 ^)^. Com o aumento da expectativa de vida da população, maior atenção deve ser dada à comunicação do idoso.

No processo de envelhecimento podem ocorrer modificações na arquitetura do sistema fonatório que constitui a presbilaringe possuindo como consequência fisiológica a alteração da produção da voz, denominada presbifonia^(^ 2 ^)^. A presbifonia, no entanto, pode ou não estar associada à presbilaringe. Ressalta-se que a presbilaringe é frequente, mas não universal nos idosos, e não deve ser confundida com características comuns da laringe^(^ 3 ^)^. Essas modificações ocorrem nas cartilagens laríngeas com a perda de elasticidade, diminuição de mobilidade das articulações laríngeas, acompanhada de atrofia das pregas vestibulares e mudanças na cobertura das pregas vocais^(^ 4 ^)^, dentre outras.

O envelhecimento natural da voz denominado presbifonia possui início e desenvolvimento que dependem da saúde física, psíquica e história de vida do indivíduo^(^ 5 ^)^. A fonoterapia vocal no envelhecimento objetiva compensar as características da presbifonia e lentificar o processo de deterioração desencadeado pela idade, melhorando a qualidade de vida do sujeito em suas atividades diárias^(^ 6 ^)^.

Dentre as técnicas vocais constantemente utilizadas na clínica fonoaudiológica, destaca-se o exercício de sons vibrantes. A técnica de vibração sonorizada de língua ou lábios é classificada como um exercício do trato vocal semiocluído, pois suaviza o contato entre as pregas vocais, aumenta a resistência vocal, otimiza o movimento muco-ondulatório, equilibra as pressões sub e supraglótica, reduz o risco de trauma fonatório, expande o trato vocal e estimula a ressonância^(^ 5 ^)^.

Entretanto, com as mudanças decorrentes do envelhecimento, pouco se sabe sobre o efeito do exercício vocal e o tempo de execução necessário na população idosa dado às mudanças anatomofisiológicas.

O interesse pela abordagem da situação de presbilaringe e presbifonia deve-se ao fato de a população de idosos ter aumentado nos últimos anos, gerando a necessidade de ampliação de estudos referentes a este grupo etário e à escassez de publicações nessa área.

O objetivo do presente estudo foi analisar e comparar os efeitos vocais imediatos da técnica de sons vibrantes sonorizados na avaliação das medidas acústicas e perceptivo-auditivas de idosas com e sem autopercepção de alteração vocal.

## MÉTODO

Trata-se de estudo clínico, quase experimental, aprovado pelo Comitê de Ética em Pesquisa - COEP, sob número de parecer 83004518.5.0000.5149. Para participarem da pesquisa todas as participantes foram informadas sobre o estudo e assinaram o termo de Consentimento Livre e Esclarecido - TCLE. Esta casuística foi composta por 53 idosas, socialmente ativas, com idades entre 60 e 70 anos.

As participantes foram selecionadas seguindo os seguintes critérios de inclusão: ser do gênero feminino; ter idade igual ou superior a 60 anos; não ter realizado tratamento fonoaudiológico para voz prévio nos últimos 12 meses, descartando-se, desse modo, a possibilidade de que a participante tivesse qualquer condicionamento vocal por treinamento com técnicas fonoterapêuticas. Enquanto os de exclusão foram: não conseguir realizar os sons vibrantes e o diagnóstico otorrinolaringológico de alteração laríngea não pertencente a um quadro de presbilaringe.

A pesquisa foi composta pela aplicação de questionário sobre dados sociodemográficos e de autopercepção vocal; julgamento perceptivo-auditivo e acústica da qualidade da voz; e avaliação laríngea, antes e imediatamente após um, três e cinco minutos de execução da técnica vocal de sons vibrantes sonorizados. Sua execução foi no Observatório de Saúde Funcional em Fonoaudiologia - OSF de uma universidade pública.

O questionário continha questões referentes a dados sociodemográficos: idade, escolaridade e aposentadoria, e o questionário de Rastreamento de Alterações Vocais - RAVI^(^ 7 ^)^.

O RAVI é um questionário curto, de baixo custo, de fácil aplicação e de resultado auto relatado que permite rastrear a alteração vocal em idosos. Ele dispõe de 10 questões, sendo que para cada uma delas existem três possibilidades de resposta (não, às vezes e sempre), numa escala tipo Likert, graduadas de zero a dois, onde: não=0, às vezes=1 e sempre=2. Para análise das respostas deve-se realizar o somatório de todas as questões respondidas. Quando o escore final estiver acima de dois pontos há indicação da presença de alteração vocal, quando este for igual ou menor que dois pontos, há indicação de ausência de alteração vocal^(^ 7 ^)^. Portanto, o RAVI deve ser utilizado como uma ferramenta de triagem para auxiliar na determinação da prevalência de distúrbios da voz em idosos^(^ 7 ^)^.

Anteriormente e durante os intervalos da realização da técnica foram coletadas amostras da vogal sustentada /a/ totalizando, dessa forma, quatro amostras. A primeira amostra equivale ao momento pré execução da técnica (M0) e a segunda após um minuto (M1). Em seguida, a técnica era executada por mais dois minutos até completar três minutos (M2) e mais dois minutos até totalizar os cinco minutos (M3) de execução da técnica de sons vibrantes sonorizados.

Todas as participantes realizaram a emissão da vogal /a/ prolongada em tempo máximo de fonação com a frequência e intensidade habituais da voz. As idosas somente iniciaram a sequência de execução dos tempos da técnica após demonstração do avaliador e pequeno treinamento com uma ou duas repetições da realização correta dos sons vibrantes sonorizados pela idosa. Antes de iniciar a coleta de M0, as idosas permaneceram em repouso vocal mínimo de 30 segundos.

Para coleta do material de fala, as participantes permaneceram sentadas em ambiente silencioso durante todo o período e foram instruídas a realizar o exercício de sons vibrantes em tempo máximo de fonação, em tom habitual sem variação, durante um, três e cinco minutos. Caso a idosa demonstrasse inabilidade para executar a vibração de língua, foi feita a substituição pela técnica de vibração de lábios (n=17). Ao longo de todo o tempo de realização da técnica vocal, as participantes puderam ingerir até 250 ml de água, para poder umidificar a cavidade oral que pode ficar ressecada devido a vibração realizada durante a execução do exercício. Vale ressaltar que a hidratação não intervém nos resultados, uma vez que a hidratação sistêmica demora algumas horas para atingir a laringe^(^ 8 ^)^.

O registro da execução do exercício foi realizado por meio do programa CSL da marca KayPentax®, modelo 4.500, acoplado em um microfone profissional unidirecional, marca Shure®, modelo SM48-LC, situado a 10 cm da falante, e em ambiente silencioso com um nível de ruído ambiental inferior a 50 dB NPS verificado através do medidor de nível de pressão sonora da marca Radio Shac®. As amostras da vogal sustentada /a/ foram utilizadas para realização das avaliações perceptivo-auditivas e acústicas.

A avaliação otorrinolaringológica foi realizada por um médico otorrinolaringologista em um hospital universitário em um período de, no máximo 15 dias, após os momentos de aplicação do questionário e de execução da técnica. A videolaringoscopia foi realizada por telescope 70°, marca Storz®, fonte de luz xênon de 300 Watts, marca Storz®, microcâmera telecam DX, marca Storz®. Solicitou-se que todas as participantes emitissem a vogal sustentada /i/ durante o período mínimo de dois segundos na mesma frequência habitual.

O médico responsável pelo exame, preencheu o Reflux Finding Score^(^ 9 ^)^ validado para o português brasileiro como Escala de Achados Endolaríngeos que pontua os achados e sinais inflamatórios laríngeos compatíveis com o refluxo laringofaríngeo (RLF) por meio do exame de videolaringoscopia. Foram avaliadas a presença de edema subglótico, obliteração dos ventrículos, eritema ou hiperemia, edema de pregas vocais, edema laríngeo difuso, hipertrofia da região interaritenóidea, granuloma/tecido de granulação, muco endolaríngeo espesso. Um resultado final maior ou igual a sete pontos nas somatória final, significa que o indivíduo tem alta probabilidade de apresentar refluxo laringofaríngeo^(^ 9 ^)^. Nenhuma participante apresentou RLF considerando o ponto de corte do instrumento. Todas as informações do exame foram registradas em um laudo médico e entregues às participantes ao final. A pesquisadora também recebeu uma cópia do exame de cada participante.

Após a coleta de todos os dados foi realizada a definição dos grupos de estudo. As idosas foram divididas em dois grupos de acordo com a classificação do RAVI^(^ 7,10,11 ^)^ e o resultado do exame laríngeo: grupo de idosas com autopercepção de alteração vocal, com sintomas vocais e exame laríngeo compatível com as características da laringe do idoso (GCAV/n=25) e grupo de idosas sem autopercepção de alteração vocal, sem sintomas vocais e com exame laríngeo normal (GSAV/n=28) (Figura 1).

**Figura 1:** Fluxograma da definição dos grupos de comparação

Para análise dos desfechos da técnica vocal foram realizadas as avaliações perceptivo-auditivas e análise acústica. Essas avaliações foram efetuadas por três fonoaudiólogas com no mínimo, cinco anos de experiência, na área de voz. As juízas receberam as vozes randomizadas em CD e foram orientadas a ouvir as gravações, quantas vezes fossem necessárias, em ambiente silencioso. As análises foram realizadas com base na emissão da vogal sustentada /a/. Para cada par de vozes as juízas foram instruídas a analisar se a segunda voz melhorou, piorou ou não modificou. Quando a avaliadora observou mudança na voz (melhora ou piora), esta foi orientada a apontar o parâmetro perceptivo-auditivo (grau geral da disfonia - G) da escala GRBASI que mais se correlacionou com a mudança na voz. Ressalta-se que as vozes foram julgadas considerando o tempo “0” como ausência total de desvio vocal. As avaliadoras não possuíam conhecimento prévio se a voz analisada era pré ou pós exercício ou a qual tempo de execução foi realizada, nem qual a idade das idosas.

Para avaliação acústica das vozes os parâmetros acústicos apurados pelo programa CSL da KayPentax® foram: frequência fundamental média - f0 em Hz, *jitter* (%), *shimmer* (%), quociente de perturbação da frequência - PPQ (%), quociente de perturbação da amplitude - APQ% (%) e proporção harmônico-ruído - PHR em dB. Para as análises foi eliminado o ataque vocal inicial da emissão /a/ e, a partir desse ponto, o tempo de 3,5 segundos foi considerado como padrão para a janela de análise, descartando-se o restante^(^ 8 ^)^. Os valores de normalidade das medidas acústicas indicados pelo manual do programa utilizado são: f0 243,97 Hz, *jitter* 0,63%, *shimmer* 1,99%, PPQ 0,36%, APQ 1,39% e PHR 0,11dB^(^ 12 ^)^.

Foi realizada também a extração das medidas cepstrais, compostas pelo CPP (*Cepstral Peak Prominence*) e CPPs (*Cepstral Peak Prominence Smoothed*) que correspondem à Transformada de Fourier em escala logarítmica^(^ 13 ^)^.

O software utilizado foi o Praat na versão 6.1.16 e os parâmetros utilizados foram os descritos por Phadke et al.^(^ 14 ^)^. A tarefa foi gravada utilizando os equipamentos anteriormente citados e o início e o fim da gravação foram excluídos para que não interferissem na análise cepstral do sinal acústico.

A análise das variáveis primeiramente foi descritiva por meio de distribuição de frequência relativa e absoluta das variáveis categóricas e de medidas de síntese numérica das variáveis quantitativas. Para avaliação perceptivo-auditiva foi realizada concordância intra-avaliadores por meio da estatística AC1, no programa R em sua versão 3.2. Os valores de concordância observados foram: avaliadora um: 0,517; avaliadora dois: 0,434 e avaliadora três: 0,458. Os valores obtidos indicam que a concordância das três avaliadoras é moderada^(^ 15 ^)^.

A partir da análise de distribuição das variáveis quantitativas por meio do teste Kolmogorov-Smirnov foram definidos os testes estatísticos para comparação dos parâmetros acústicos dos grupos entre os tempos de execução da técnica (Teste Anova para medidas repetidas, Teste de Friedman e Teste Wilcoxon). Os testes estatísticos utilizados na associação do julgamento perceptivo-auditiva entre os grupos GCAV e GSAV foram o Exato de Fisher e o Qui-Quadrado de Pearson. Para todos os testes foi considerado o nível de significância de 5%.

## RESULTADOS

A maioria das participantes do presente estudo possui entre 60 e 70 anos de idade (60,37%), são alfabetizadas (75,47%) e aposentadas (88,67%). Na Tabela 1 observa-se uma predominância de disfonia de grau moderado nos dois grupos de comparação, segundo o julgamento perceptivo-auditivo das especialistas.

**Tabela 1:** Descrição do grau geral da disfonia na avaliação perceptivo-auditiva dos grupos pré realização da técnica de sons vibrantes

**Grupos**	**Grau Geral**	**n (%)**
GCAV (n=25)	Neutra	3 (12,0)
	Leve	5 (20,0)
	Moderado	13 (52,0)
	Intenso	4 (16,0)
GSAV (n=28)	Neutra	3 (10,7)
	Leve	10 (35,7)
	Moderado	12 (42,9)
	Intenso	3 (10,7)

Na avaliação perceptivo-auditiva houve uma predominância de melhora da voz após a realização da técnica em todos os tempos, com maior frequência após a execução por um minuto. Ao comparar os grupos (Tabela 2) quanto à autopercepção vocal das idosas não houve diferença estatisticamente significante na associação da melhora, piora ou inalteração das vozes, ou seja, a mudança na voz após a execução da técnica em todos os tempos não foi diferente entre as idosas com e sem autopercepção de alteração vocal.

**Tabela 2:** Associação da avaliação perceptivo-auditiva entre GCAV e GSAV segundo o tempo de execução da técnica

**Avaliação perceptivo-auditiva**		**Amostra total n (%)**	**Grupos**		**Valor P** 1
			**GCAV (n=25)**	**GSAV (n=28)**	
			**n (%)**	**n (%)**	
G após 1 minuto	Melhorou	30 (56,6%)	13 (52,0)	17 (60,7)	0,747*
	Piorou	9 (17,0%)	4 (16,0)	5 (17,9)	
	Inalterada	14 (26,4%)	8 (32,0)	6 (21,4)	
G após 3 minutos	Melhorou	25 (47,1%)	12 (48,0)	13 (46,4)	0,828**
	Piorou	13 (24,5%)	7 (28,0)	6 (21,4)	
	Inalterada	15 (28,3%)	6 (24,0)	9 (32,1)	
G após 5 minutos	Melhorou	23 (43,4%)	13 (52,0)	10 (35,7)	0,416**
	Piorou	13 (24,5%)	6 (24,0)	7 (25,0)	
	Inalterada	17 (32,0%)	6 (24,0)	11 (39,3)	

1 Significância (p) ≤ 0,05;

* Exato de Fisher;

** Qui-Quadrado de Pearson

**Legenda:** G = grau geral da GRBASI; GCAV = grupo com autopercepção de alteração vocal; GSAV = grupo sem autopercepção de alteração vocal

Na Tabela 3 realizou-se comparação entre os parâmetros acústicos e os tempos de execução da técnica de sons vibrantes sonorizados. No GCAV foi possível observar que houve associação com significância estatística apenas entre APQ (p=0,022). Para verificar em qual par(es) ocorreu a diferença foi realizado o teste Wilcoxon que revelou diferença entre os tempos M0xM1 (p=0,013). As demais associações não revelaram resultados com significância estatística.

**Tabela 3:** Comparação dos parâmetros acústicos do grupo com autopercepção de alteração vocal (GCAV) entre os tempos de execução da técnica (n=25)

**Variáveis**	**Momento**	**Média**	**Mediana**	**DP**	**Valor P**
F0 (Hz)^1^	M0	188	186,06	31,67	0,878
	M1	191,76	189,72	43,88	
	M2	190,12	191,89	43,19	
	M3	191,76	194,85	44,33	
APQ^2^	M0^3^	5,37	4,53	3,06	0,022*
	M1^3^	4,02	3,84	2,34	
	M2	4,8	4,02	3,04	
	M3	4,97	3,8	4,43	
PPQ^2^	M0	1,38	1,01	1,02	0,123
	M1	0,94	0,65	0,82	
	M2	1,01	0,82	0,74	
	M3	1,17	0,86	1,09	
NHR^2^	M0	0,23	0,19	0,1	0,231
	M1	0,18	0,17	0,06	
	M2	0,18	0,17	0,05	
	M3	0,21	0,16	0,1	
*SHIMMER* ^2^	M0	7,7	6,79	4,53	
	M1	5,74	4,84	3,84	0,113
	M2	6,57	5,02	5,47	
	M3	6,74	5,46	5,24	
*JITTER* ^2^	M0	2,37	1,88	1,69	0,311
	M1	1,87	1,14	1,67	
	M2	1,72	1,43	1,2	
	M3	2,02	1,33	1,88	
CPP^1^	M0	26,87	27,35	3,37	0,369
	M1	27,53	27,79	2,76	
	M2	27,48	27,49	3,05	
	M3	27,4	27,76	3,2	
CPPs^1^	M0	15,98	15,96	2,84	0,124
	M1	16,87	16,96	2,32	
	M2	16,53	16	2,9	
	M3	16,62	16,67	2,61	

1 Teste Anova para medidas repetidas;

2 Teste de Friedman;

3 Teste Wilcoxon;

* Significância (p) ≤ 0,05

**Legenda:** DP = desvio padrão; F0 = frequência fundamental; Hz = hertz; APQ = quociente de perturbação da amplitude; PPQ = quociente de perturbação da frequência; NHR = proporção harmônico-ruído; CPP = *cepstral peak prominence*; CPPs = *cepstral peak prominence smoothed*; M0 = avaliação antes da realização da técnica; M1 = após 1 minuto de execução da técnica; M2 = após 3 minutos de execução da técnica; M3 = após 5 minutos de execução da técnica

As mesmas análises da Tabela 3 foram realizadas na Tabela 4, agora para o GSAV. Em sua análise observa-se que não houve associação com significância estatística em quaisquer das análises realizadas.

**Tabela 4:** Comparação dos parâmetros acústicos do grupo sem autopercepção de alteração vocal (GSAV) entre os tempos de execução da técnica (n=28)

**Variáveis**	**Momento**	**Média**	**Mediana**	**DP**	**Valor P** *
F0 (Hz)^1^	M0	177,62	182,55	32,73	0,262
	M1	186,45	189,53	29,63	
	M2	179,83	183,01	36,44	
	M3	184,64	185,49	28,34	
APQ^2^	M0	4,39	3,64	2,41	0,478
	M1	3,16	1,76	1,4	
	M2	4,13	2,94	2,85	
	M3	3,54	2,95	1,72	
PPQ^2^	M0	0,99	0,67	0,89	0,887
	M1	0,69	0,53	0,5	
	M2	0,83	0,61	0,74	
	M3	0,82	0,52	0,73	
NHR^2^	M0	0,19	0,16	0,08	0,219
	M1	0,16	0,14	0,05	
	M2	0,17	0,15	0,07	
	M3	0,17	0,15	0,06	
*SHIMMER* ^2^	M0	6,06	5,3	3,6	0,372
	M1	4,25	3,79	1,86	
	M2	5,89	3,97	4,29	
	M3	4,94	4,05	2,65	
*JITTER* ^2^	M0	1,76	1,2	1,5	0,663
	M1	1,22	0,96	0,81	
	M2	1,47	1,13	1,26	
	M3	1,41	0,87	1,13	
CPP^1^	M0	27,51	27,5	3,02	0,212
	M1	28,51	28,48	1,98	
	M2	28,36	28,54	2,93	
	M3	28,65	29,2	2,63	
CPPs^2^	M0	16,49	16,57	2,67	0,096
	M1	17,65	17,5	1,71	
	M2	17,29	17,01	2,69	
	M3	17,64	18	2,3	

1 Teste Anova para medidas repetidas;

2 Teste de Friedman;

* Significância (p) ≤ 0,05

**Legenda:** DP = desvio padrão; F0 = frequência fundamental; Hz = hertz; APQ = quociente de perturbação da amplitude; PPQ = quociente de perturbação da frequência; NHR = proporção harmônico-ruído; CPP = *cepstral peak prominence*; CPPs = *cepstral peak prominence smoothed*; M0 = avaliação antes da realização da técnica; M1 = após 1 minuto de execução da técnica; M2 = após 3 minutos de execução da técnica; M3 = após 5 minutos de execução da técnica

## DISCUSSÃO

No presente estudo houve predomínio de disfonia de grau moderado, independente do grupo averiguado, considerando a avaliação perceptivo-auditiva das especialistas. Este achado corrobora a pesquisa^(^ 16 ^)^ que avaliou de forma perceptivo-auditiva as características vocais de idosos institucionalizados com intuito de verificar se essas características iriam interferir no processo de comunicação e no padrão de fala. Os autores concluíram que o grau de alteração vocal predominante foi o grau moderado e que existem alterações nos parâmetros referentes à voz decorrentes da idade. Sabe-se que é esperado o envelhecimento natural da voz com o decorrer da idade^(^ 5 ^)^, sendo que o resultado do estudo reforça a ausência de correlação entre a presença de presbifonia e presbilaringe.

A avaliação perceptivo-auditiva do especialista se difere da autopercepção de alteração vocal do idoso. Um estudo avaliou a voz de um grupo de idosos e relacionou a qualidade vocal com o grau de alteração da voz. Essa associação foi realizada por meio da avaliação perceptivo-auditiva e de uma escala de autoavaliação vocal. Os participantes avaliaram a própria voz com um impacto positivo na vida particular, profissional e social. Os autores notaram que os resultados obtidos foram positivos mesmo nas vozes com qualidade vocal alterada havendo predomínio do impacto bom e ótimo na autoavaliação vocal dos idosos^(^ 17 ^)^.

O idoso com presbifonia, no entanto, pode apresentar a voz rugosa, fraca, soprosa, com redução do tempo máximo de fonação e da extensão fonatória. Tais características, esperadas na senescência, podem levar a uma ininteligibilidade de fala e a um impacto negativo na qualidade de vida^(^ 18 ^)^. A avaliação vocal do GSAV, composto por idosas sem relato de sintomas vocais, mostrou presença de alteração vocal em quase todas as participantes. A falta de percepção do idoso quanto aos sintomas vocais pode estar relacionada ao fato de algumas pessoas possuírem boa imagem formada sobre sua voz, outros nunca terem pensado no assunto no decorrer da vida e alguns por considerarem os sintomas vocais como naturais do envelhecimento, não passíveis de melhora. Salienta-se que o impacto da alteração vocal na qualidade de vida não foi investigado nesse estudo, sendo um importante instrumento na avaliação multidimensional da voz.

Pesquisas realizadas^(^ 19-21 ^)^ com o exercício de vibração de língua foram executadas na população adulta com e sem alteração vocal. Compreende-se a existência de diferenças na anatomia do trato vocal dos idosos e dos adultos^(^ 8,16 ^)^. No presente estudo, apesar da ausência de significância estatística intergrupos em todos os tempos de execução da técnica vocal verificou-se melhora na qualidade vocal das idosas. Pode-se dizer que o efeito da técnica foi positivo nos três tempos avaliados em ambos os grupos, porém houve redução da melhora à medida que o tempo de execução aumentava. Esse resultado foi contraditório ao esperado, visto que para uma melhor adaptação fisiológica sobre a condição laríngea do idoso, este poderia necessitar de um maior tempo de estímulo de exercício vocal. Destaca-se que quase todas as idosas participantes apresentaram laringe normal. As medidas respiratórias não foram avaliadas, porém podem ter interferido no equilíbrio da dinâmica de produção vocal com a piora na execução da técnica ao longo do tempo.

Em revisão de literatura^(^ 22 ^)^ sobre evidências de hipertrofia muscular em pessoas idosas por meio do treinamento de resistência os autores mostraram que é possível melhorar a massa muscular em treinamento de resistência em pessoas idosas, sobretudo os mais longevos, desde que os exercícios apresentem a dose correta: intensidade, volume e carga apropriada. Pouco se sabe sobre essa dose no tratamento fonoaudiológico de voz para idosos. Para o sucesso da terapia vocal em idosos também são necessárias mudanças na intensidade vocal, no uso da musculatura, e no suporte respiratório. Além do mais, é de extrema importância a manutenção dessas mudanças no sistema fonatório^(^ 23 ^)^.

A literatura sugere que o exercício de sons vibrantes proporciona resultados positivos a partir de três minutos e que o tempo de execução ideal do exercício são por três minutos, em ambos os sexos; ou três séries de 15 repetições com intervalos de 30 segundos, em mulheres adultas^(^ 24 ^)^. Outro estudo, entretanto, propõe o tempo de cinco minutos para os homens adultos^(^ 20 ^)^. Em crianças disfônicas, contudo, verificou-se que o exercício de vibração sonorizada de língua não promove melhora em nenhum tempo de execução (um minuto, três minutos, cinco minutos e sete minutos)^(^ 25 ^)^.

Em estudo^(^ 5 ^)^ realizado com 33 indivíduos com média de idade de 75 anos a fim de observar o efeito imediato da realização do exercício do sopro sonorizado com o trato vocal semiocluído na voz do idoso após um minuto de exercício, observou-se que este exercício produz efeitos positivos imediatos na qualidade vocal de idosos, observados na análise perceptivo-auditiva. Tal achado corrobora os achados desta pesquisa quanto ao tempo de execução da técnica. Ressalta-se que em ambos os estudos os exercícios são de trato vocal semiocluído e que após a execução destes por um minuto, a emissão vocal do idoso foi melhor que a apresentada por ele em situação habitual.

Destaca-se que esta pesquisa analisou os efeitos imediatos da técnica vocal de sons vibrantes sonorizados. Considerando o processo de acompanhamento longitudinal da fonoterapia, a literatura descreve, para a população idosa, o número de sessões, frequência e duração do tratamento dos distúrbios da voz, entretanto, os resultados são variáveis^(^ 26 ^)^. Existem pesquisas que enfatizam o benefício do tratamento intensivo para a população idosa^(^ 27 ^)^. Estima-se que este tipo de abordagem intensiva propicie o aprendizado motor, mudança de comportamento e melhor desempenho da musculatura, em tempo inferior ao da terapia tradicional^(^ 28 ^)^.

Quanto aos resultados da análise acústica houve redução das medidas de APQ, PPQ, PHR, *shimmer* e *jitter* após o primeiro minuto de vibração sonorizada em ambos os grupos estudados, porém com significância estatística somente para APQ no GCAV. Embora os valores encontrados diminuam após o primeiro minuto pode-se dizer que estes se encontram alterados uma vez que a padronização do programa utilizado é para mulheres jovens de 20 a 45 anos^(^ 29 ^)^. A falta de valores de referência das medidas analisadas para população idosa dificulta a interpretação desses dados.

No que se refere ao quociente de perturbação da amplitude - APQ observa-se redução nos valores após a execução dos sons vibrantes sonorizados indicando, assim, maior estabilidade na amplitude de vibração das pregas vocais, ciclo a ciclo, e consequentemente melhor controle fonatório. A diminuição do APQ refere-se à passagem e controle do ar transglótico a cada ciclo glótico sugerindo, desse modo, menor escape aéreo transglótico^(^ 30 ^)^. Esse resultado indica a melhora na emissão da vogal sustentada das idosas, principalmente para aquelas com autopercepção de alteração vocal. A redução de algumas medidas acústicas reforça o começo de um melhor efeito da técnica na qualidade vocal das idosas de ambos os grupos após 1 minuto, com uma certa estabilidade dos valores após esse tempo.

A análise das medidas cepstrais em função do tempo de exercício não demonstrou significância estatística apesar de ser percebido um incremento nos valores obtidos nas medidas de CPP e CPPs, em ambos os grupos. Portanto, não é possível estabelecer relação temporal entre tempo de execução da técnica empregada com a melhora dos parâmetros vocais de alta correlação com as medidas cepstrais, como a rugosidade e a soprosidade^(^ 3 ^)^. Por outro lado, deve-se considerar que um dos sinais do envelhecimento é a deterioração das fibras musculares^(^ 4 ^)^. Portanto, supõe-se que a realização progressiva dos exercícios possa ter alcançado a fadiga muscular e, por conseguinte, não haver melhora funcional progressiva como demonstrado em estudos realizados com indivíduos mais jovens^(^ 20,24 ^)^.

Verifica-se que é preciso considerar a idade e o gênero para prescrição do tempo de execução da técnica de vibração sonorizada visando um resultado mais imediato e positivo na voz. Idosos podem ser beneficiados com programas de promoção da saúde vocal por meio de orientações sobre comportamento vocal e treinamento de técnicas vocais para redução dos sintomas e melhora na qualidade vocal. A fonoterapia vocal no envelhecimento objetiva compensar as características da presbifonia, otimizar os aspectos comunicativos, e lentificar o processo de deterioração causado pela idade, melhorando a qualidade de vida do sujeito em suas atividades diárias^(^ 8 ^)^.

Os resultados da avaliação perceptivo-auditiva e da análise acústica no presente estudo mostram o benefício da técnica de vibração sonorizada para a voz das idosas, como destaque ao GCAV após a execução por um minuto. Contudo, não é possível afirmar qual o tempo ideal de realização da técnica para melhorar a qualidade vocal de idosas.

Como limitação do presente estudo, a não realização de cálculo amostral, tamanho da amostra reduzido e o fato da amostra ser de conveniência impede a generalização dos resultados. A decisão de comparar os grupos considerando a autopercepção de alteração vocal pelas idosas junto com o resultado do exame laríngeo permitiu mostrar a complexidade na realização de pesquisas sobre voz com a população idosa uma vez que os idosos podem estar acometidos por diversas doenças crônicas, fazer uso de medicações e possuir comportamentos vocais inadequados ao longo da vida, possíveis fatores etiológicos para disfonia. Não foi avaliado se a realização de variações da técnica de sons vibrantes sonorizados (lábios e língua) pelas idosas pode ter interferido de alguma forma nos resultados.

Sabe-se que a disfonia é multicausal e para o estabelecimento de sua etiologia na população idosa deve-se levar em consideração as peculiaridades advindas do processo de envelhecimento. Embora haja o envelhecimento natural da voz do idoso é de extrema importância a investigação clínica dos sintomas vocais em idosos por meio da avaliação completa. Sintomas vocais em idosos precisam ser valorizados e avaliados, pois podem ser sinais de doenças como: doença do refluxo laringofaríngeo, tumor de laringe e doenças neurodegenerativas em estágios iniciais. O diagnóstico de presbifonia deve considerar a avaliação perceptivo-auditiva, a análise acústica, a autopercepção vocal e a avaliação laríngea de idosos.

Convém ressaltar que as idosas estudadas nesta pesquisa não apresentaram RLF. A composição do ácido pode prejudicar a laringe e gerar inflamação, fato que diminui a eficácia de comunicação do indivíduo, podendo causar ou agravar os distúrbios da voz^(^ 1 ^)^, sendo importante se investigar na população idosa.

Dessa forma, os resultados do presente estudo poderão auxiliar e orientar a prática clínica fonoaudiológica na população idosa uma vez que o uso da técnica de sons vibrantes sonorizados atingiu resultados positivos e este exercício pode ser usado para melhorar a qualidade vocal da mulher idosa independente da autopercepção de alteração vocal.

Estudos que averiguem os efeitos do tempo de exercícios vocais na população do gênero masculino também são necessários, a fim de compreender os resultados funcionais das pregas vocais nos diferentes gêneros e se obter os tempos de execução de técnicas vocais. Pesquisas futuras que avaliem os efeitos imediatos da execução da técnica de sons vibrantes sonorizados com variação de frequência também são importantes, considerando os aspectos de treinamento funcional que objetivam o fortalecimento e a mobilidade da musculatura na população idosa.

Cabe ressaltar que o fonoaudiólogo, enquanto clínico vocal deve levar em consideração as recomendações acerca das prescrições dos exercícios, especialmente na população idosa uma vez que esta necessita de conhecimento a respeito da fisiologia do envelhecimento. Portanto, é necessário analisar os efeitos imediatos e em longo prazo dos exercícios, considerando o tempo de execução e o número de repetições diárias das técnicas vocais.

## CONCLUSÃO

Idosas apresentam elevada presença de alteração vocal quando considerado o julgamento perceptivo-auditivo da voz por especialistas. Apesar dos resultados indicarem um predomínio da melhora na voz das idosas após a execução da técnica de sons vibrantes nos diversos tempos de execução, não houve evidência científica quanto ao tempo ideal para se obter um melhor efeito na voz das idosas. Destaca-se a resposta perceptivo-auditiva positiva em ambos os grupos investigados após o primeiro minuto.
